# Development and evaluation of a stroke research Public Patient Involvement Panel

**DOI:** 10.12688/hrbopenres.13838.1

**Published:** 2024-04-24

**Authors:** Siobhán O'Reilly, Aoife Whiston, Eva Corbett, Amy O'Mahony, Molly X Manning, Pauline Boland, Katie Robinson, Rose Galvin, Joanna M Allardyce, Mike Butler, Jim Bradley, Jon Salsberg, Margaret O'Connor, Patricia Pond, Eva Murphy, Liam G Glynn, Nora Cunningham, Edel Hennessy, Sara Hayes

**Affiliations:** 1 School of Allied Health, University of Limerick, Limerick, County Limerick, Ireland; 2 Health Research Institute, University of Limerick, Limerick, County Limerick, Ireland; 3 Public and Patient Involvement Research Unit, Health Research Institute, University of Limerick, Limerick, County Limerick, Ireland; 4 Ageing Research Centre, Health Research Institute, University of Limerick, Limerick, County Limerick, Ireland; 5 University of Limerick, Limerick, County Limerick, Ireland; 6 School of Medicine, University of Limerick, Limerick, County Limerick, Ireland; 7 University Hospital Limerick, Dooradoyle, County Limerick, Ireland

**Keywords:** Public and patient involvement, stroke, stakeholder, stroke research, PPI

## Abstract

**Background:**

Public and patient involvement (PPI) is important in stroke research to ensure that research conducted reflects the priorities and needs of people after stroke. Several factors have been found to affect PPI, including location of the research and time requirements for participation. The incidence of stroke is rising, and can result in symptoms including fatigue, depression, and physical/cognitive impairments.

**Aims:**

1) Describe the development of a PPI advisory group and a healthcare professional advisory group for stroke rehabilitation research and 2) to explore the perspectives of the members of the PPI groups on being involved in the research process.

**Methods:**

A stakeholder panel consisting of up to 20 people with stroke, members of the public and healthcare professionals will be formed. A pragmatic purposive sampling technique using snowball sampling will be used to recruit members. The PPI panel will meet four times and will be supported by the guidelines developed from the INVOLVE framework. The PPI panel will be involved as co-researchers in the conceptualisation of future stroke rehabilitation research, the delivery of such studies, the analysis and dissemination of findings. Following the development of the panel, we will conduct semi-structured focus groups to collect qualitative data, examining the perspectives of members. Separate focus groups will be held for people with stroke, family members/cares, and healthcare professionals/researchers. Data will be transcribed and analysed using Braun and Clarke’s Reflexive Thematic Analysis. This will result in a set of themes and subthemes describing participants' opinions and experience of being on a PPI panel in stroke rehabilitation research.

**Conclusions:**

PPI is an essential part of research in stroke. Stakeholders can provide key insights into the research processes. The results of this qualitative study will provide insight into the barriers and enablers of their participation in PPI in stroke rehabilitation research.

## Introduction

Drawing on the diverse perspectives and lived experience of people living with health conditions, their families and caregivers, and health professionals, is increasingly recognised as an important dimension of strengthening the quality of health and social care research ^1^. There is particular emphasis on the need to consider Public & Patient Involvement. in health and social and is a key pillar of high-quality research internationally. The National Institute for Health Research (NIHR) advisory group, INVOLVE defines PPI as “research being carried out 'with' or 'by' members of the public rather than 'to', 'about' or 'for' them”. The term ‘public’ is used to include patients, potential patients, carers, and anyone who uses health and social care services or represents service users.

PPI is important in stroke research to ensure that people with stroke receive evidence-based, patient-centred, and holistic management of their condition. As the population is ageing, a 70% increase in the absolute number of incident strokes has occurred between 1990 to 2019 ^2^. The Irish National Audit of Stroke’s National Report in 2020 found that stroke is the leading cause of acquired adult disability in Ireland. Aside from increased mortality rates, there are several burdensome consequences of stroke that can reduce a person’s quality of life ^3^. Post-stroke fatigue can be a debilitating symptom, with two-fifths of people with stroke reporting it as their worst symptom ^4^. One systematic review highlighted that about 24% of people had anxiety up to 12-months post-stroke ^5^, impacting participants’ adherence to rehabilitation services. Moreover, roughly 2/3 of people post-stroke have communication problems, with 1/3 having aphasia ^6, 7^. Cognitive impairment and communication deficits can affect quality of life, which a significant proportion of people with stroke can develop ^8– 10^. The pooled prevalence of dementia among people with stroke in hospital-based studies was 26.5% ^11, 12^. The economic burden of stroke is dependent on several factors including severity, type of stroke, underlying cause(s), and patient personal factors. It has been proposed that post-stroke rehabilitation can cost approximately €29,484 per patient in the first year ^13^. As the survival rate of stroke has increased over the past two decades rehabilitation programmes following the acute stage are required to manage the on-going symptoms of this condition ^14, 15^.

PPI in stroke research to date has been conducted by individuals joining an advisory group, helping to select research and design research projects, and aiding in ensuring understandable research results ^16^. The individuals involved in PPI activities for stroke research should include people who have had a stroke, caregivers for people with stroke, family members of people who have experienced a stroke and any other people who use health and social care services ^17^. Additionally, inclusion of harder-to-reach groups is beneficial and important in the development of a PPI panel ^18^.

Incorporating patients and the public into stroke research has many advantages ^19^. On a personal level, active PPI in stroke studies can foster the formation of supportive and trusting connections among PPI members with researchers ^20^. Additionally, it can offer caregivers valuable opportunities for respite from their caregiving responsibilities while also serving as a catalyst for intellectual engagement and stimulation among patients ^21^. Several barriers have been found to affect PPI, including location of the research in relation to the participants, time requirements for participation, and people with stroke’s ability to concentrate and comprehend information ^21^. To overcome these barriers, activities or meetings should be held in accessible environments for people with disabilities, have subsided and easy transport access and run by lay or research member(s) with adequate training to facilitate these ^21, 22^. Additionally, funding agencies, such as the National Institute for Health Research, emphasise funding applications and research projects that incorporate public and patient involvement ^23^. PPI can support implementation research by setting priority areas and shaping the research question ^24^.

This study hopes to document the process of setting up a PPI panel for stroke and explore the experiences of the panel members. It will add to the existing evidence base, where some previous case studies have been reported wherein people with stroke were involved as co-researchers ^25– 28^. PPI members will be recruited, trained, and supported in contributing to this project so that recommendations can be made for the optimisation of recruitment and support for people with stroke in PPI panels. We will examine peoples’ perspectives and experiences on being involved as co-researchers in a PPI advisory group.

The aim of this study is to: 1) describe the development of a PPI advisory group for current and future stroke rehabilitation research and 2) to explore the perspectives of the members of the PPI group on being involved in the research process.

## Methods

This study will be reported in line with the Consolidated Criteria for Reporting Qualitative Research (COREQ) checklist ^29^.

### Development of the PPI panel

The current team of researchers have already begun the development of a small PPI group, including two people with stroke and a patient advocate member of the Irish Heart Foundation. We aim to further develop this PPI panel by including more people with stroke, family carers, healthcare professionals and stroke researchers. To recruit people to the PPI group, a pragmatic purposive sampling technique using snowball sampling will be used. Inclusion criteria for panel members will include adults aged 18 years and over with stroke, family members or carers of people with stroke, and healthcare professionals who work with people with stroke and researchers and academics who work in stroke rehabilitation research at the University of Limerick. It is hoped that these panel members will join the new panel and invite other potential people to join. The researchers will contact known members or organisers in patient organisations including the Irish Heart Foundation, Age Action Ireland, Headway, community groups, and family carer organisations to recruit potential PPI participants. Based on the recommendations of Gilfoyle *et al*. ^23^, a social media recruitment campaign will be implemented. Recruitment from a clinical setting can be a barrier for some, therefore social media recruitment may help to reach those in less represented groups such as members of minority groups. Moreover, it was recommended that working with existing research partners is essential in deciding on recruitment strategies ^23^. Therefore, discussions were held with members on the research team who have extensive experience in PPI.

The ‘level of involvement’ model comprises three stages: namely ‘consultation’, ‘collaboration’ and ‘user-control’ ^21^. ‘User-control’ is the highest level of involvement and it comprises the patients, members of the public, caregivers and or/ family members organising and deciding on the research agenda and recruiting researchers to work alongside them ^21^. This is the level that will be used for this PPI panel. Members of the public will be recruited through posters and newsletters circulated on social media and relevant organisations, such as University Hospital Limerick and the Irish Heart Foundation. A press release will be published on the University of Limerick’s website and affiliated social media platforms, such as Twitter and LinkedIn. It is hoped that following this, word of mouth will inform wider members of the pubic of the PPI panel formation. We will liaise closely with our clinical and advocacy partners to purposefully draw on as diverse a population as possible in terms of sociodemographic (e.g. gender, rural versus urban dwelling, living alone/with others), clinical (e.g. severity of stroke, presence of aphasia, level of mobility) backgrounds, to promote inclusivity among the PPI panel members and will be guided by the recommendations outlined by both Concannon, Meissner ^30^ and Gilfoyle, Melro ^23^., Concannon *et al.* ^30^ recommend using the 7Ps framework to identify key groups for inclusion in the panel. These are: patients and the public; providers (carers and family); purchasers (healthcare professionals); payers; policy makers; product makers; principal investigators. The authors recommend this as a flexible approach, therefore the relevant members for the purpose of this study will exclude payers, policy makers and product makers. It is important to ensure a balance between the included groups, ensuring patients and the public are strongly represented. Concannon *et al*. ^30^ do not directly address the issues of representation of minority or underrepresented groups, or the ethical considerations involved in recruitment. This may include researchers’ duty of care towards vulnerable PPI contributors and the vulnerability that may be created from participants serving on the same panel as their healthcare providers. The recruitment strategies outlined above by Gilfoyle *et al*. ^23^ will be used to address these issues.

Interested individuals will be encouraged to contact the postdoctoral researcher (A.W.) to obtain more information on becoming a PPI panel member. A total of approximately 20 members purposively sampled will be accepted onto the PPI panel. This will involve identifying key groups to target ^31^, which will be guided by Concannon, Meissner ^30^. It is anticipated that approximately five researchers and health and social care professionals and five carers will join the PPI panel. A further 10 people who have had a stroke are expected to contribute to the panel.

Each invited member (people with stroke, carer/family member, healthcare professional) will be given an information letter and a consent form to be signed. Opportunities will be given to clarify, or negotiate, aspects of joining the PPI panel. Transportation and attendance aspects will be discussed, and alternatives will be offered if required. The research team will answer all queries that prospective members may have. Emergency contact details will be collected from each participant.

### Framework to be used in the development of the PPI panel

The formation of a PPI panel for stroke research will be supported by the guidelines developed from the INVOLVE framework. This will ensure that all members of the PPI panel are treated ethically and respectfully. This framework has outlined standards that must be met when involving the public or patients in research. This comprises of inclusive opportunities (ensuring that becoming a member is accessible and available to the target research group); working together (members of the public should be able to work with the researchers, rather than work for); support and learning (training and learning opportunities should be provided for panel members); communication (communication and language used should be easily understandable by the general population and alternative communication methods should be used when required).; impact (findings from PPI should be disseminated to the public to share the knowledge); and governance (members of the PPI panel should be involved in leadership and decision-making roles). These standards have been designed to encourage researchers to adapt the involvement of PPI in their research and to ensure learning and reflection takes place for both PPI members and researchers alike ^32^.

### Panel involvement

This study will consist of four in-person meetings per year for a period of two hours which will take place between September 2023 and September 2024. Online meetings will be offered to participants as appropriate and according to the participant’s preference. To ensure PPI members feel comfortable to share their opinions and experiences, separate meetings with be held with people with stroke and their caregivers, and the healthcare professionals and researchers. Meetings held in person will be carried out in disability-friendly environments that can be easily navigated and have close access to public transport. The locations will be wheelchair friendly with either ramps or elevators. Clear instructions on finding the location of in-person meetings and details on relevant public transport routes will be emailed to the panel prior to each meeting. Potential sensory deficits will be catered for, where possible, using rooms with minimal noise interferences, voice projection, and written information will be printed with large, visible text. To address the potential difficulties with communication among PPI panel members, we will engage the expertise of an expert Speech and Language Therapist to inform the development of all materials used and the operationalisation of the meetings. Plain English will be used to make sure that all members can understand the content of the meetings, regardless of their level of understanding ^26^. For those who are unable to read the information, there will be people available to support these people and read the information aloud or present it in an alternative way to ensure understanding. PPI members will be allowed to increase or reduce their involvement in the meetings. The aims of each meeting will be clearly explained to members, with the role of each panel members discussed to manage expectations. Participants will be offered training through the PPI Ignite summer school free of charge.

The Initial meeting will introduce the panel to each other and will allow for rapport to be built between the core research team and the panel. A terms of reference document will be drawn up and reviewed by all for input. Consent will be gained from all members. Minutes will be taken during the group meetings and interviews. PPI members will be given tasks to compete and will be invited to share their opinions and suggestions. Open discussion will be encouraged and will be guided by the PI. Each stakeholder panel meeting will have a different focus. Sessions will include the following: discussion of proposed research studies, presentation of materials for participants in studies e.g., Questionnaire items, discussion about the feasibility of proposed studies, dissemination of study findings to PPI stakeholders, reflective discussion about the stakeholder panel process. Each meeting will begin with an introductory period to allow members to get to know or socialise with each other. Activities used during PPI panel meetings among co-researchers will include icebreakers and energisers, brainstorming using flipcharts, post-its, drawing and discussion, and spectrum mapping. The PPI panel will be involved as co-researchers in the conceptualisation of current and future stroke rehabilitation research, the delivery and design of research studies and the analysis and dissemination of research findings. There will be opportunities for panel members to co-present findings at public events and conferences. Meetings will last no more than two hours. Lunch will be provided for all members and members will be provided with vouchers to reimburse them for their time.

### Design of the qualitative study

To address the second aim of this research, we will use qualitative descriptive methods to examine the perspectives of the PPI panel members on their participation as co-researchers in the PPI panel. This will involve a qualitative study following the four meetings, wherein each member of the PPI panel will be invited to participate in a focus group interview following one year of PPI panel involvement. Participation is voluntary and members of the panel can opt out of the focus groups while remaining a PPI member. Focus groups will be facilitated with the three stakeholder groups individually in the first instance: (1) people with stroke, (2) healthcare professionals, physicians, and patient advocates and (3) family members and carers. The focus groups will be facilitated by SO’R, and SH and it is estimated that one focus group with 4–6 participants each per stakeholder group will be held, culminating in one combined focus group involving members from all stakeholders to determine consensus among groups.

The interview guides for this can be found in Appendix A. During these interviews, stakeholders will be invited to reflect on, and discuss, their experiences while participating in the PPI meetings. Questions will be objective and leading language will be avoided. These meetings will be audio-recorded and run for approximately one hour. There will be opportunities for the participants to make changes to their answers during the interviews for more meaningful feedback. A debriefing session will be held to ensure the panel are happy with their involvement and the interview.

### Qualitative data analysis

Data transcription, analysis, interpretation, and write-up will be carried out by the first author (SO’R) and the lead author (SH). The focus group and interviews will follow an interview guide of questions pertaining to members’ experiences and opinions of the PPI group. These meetings will be audio-recorded and transcribed verbatim. Identifying information, including names, will be removed to ensure anonymity. The audio recordings will then be destroyed. Pseudo-anonymised transcripts will be kept in a folder on password protected computers and will only be shared with necessary members of the core research team for analysis.

This study will use a qualitative approach with reflexive thematic analysis of data ^33^. the Guidance for Reporting Involvement of Patients and the Public 2 (GRIPP2) checklist will be used as a guide for reporting patient and public involvement ^34^. The GRIPP2 reporting guideline is an evidence-based checklist specifically for reporting PPI research. The implementation of this, along with the co-production of findings with the panel members and researcher reflexivity, will bolster the accuracy and quality of published findings from the PPI meetings.

Reflexivity will be at the core of this analysis, where the authors will reflect on their own experiences, knowledge, and biases ^35^. Braun and Clarke’s six-step reflexive thematic analysis ^36, 37^ will be used to analyse the transcripts. This method of analysis is flexible and allows for large datasets to be analysed while providing a rich account of the data. The transcripts are analysed under the following six steps: familiarisation of the data, coding, theme generation, searching, reviewing, and defining themes. The reflexivity will help the authors to acknowledge their biases and how these may affect the analysis of the interviews. Peer debriefing sessions will be held with other members of the research team and participants from the stakeholder panel. The aspect of reflexivity will allow for understanding and acknowledgement of bias. Themes and subthemes will be generated by the authors from the coding tree following this process ^35^.

Transcripts will be coded and stored using NVivo (Version NVT20) In situations where members of the research team are not using NVivo, paper copies and highlighters will be used for coding. These paper copies will be stored in accordance with the University of Limerick’s General Data Protection Regulation guidelines. Members of the stakeholder panel will be invited to participate in the analysis process and will aid in creating lay summaries for public consumption. 

### Ethical considerations

Ethical approval is required for this study as data will be extracted and analysed from the meetings and interviews. PPI formation itself, however, does not require approval. Ethical approval has been granted by the Education and Health Science (EHS) ethics committee in the University of Limerick (2022_02_15_EHS) on 02/10/2023.

### Plans for dissemination of study outcome

The findings will be disseminated via open access academic journals and presented at academic conferences. Free public events will be held on campus at the University of Limerick where results of the study will be made available to people who have had a stroke, carers, and health and social care professionals. The findings will be shared on social media sites to reach as many people as possible. This information will be shared as infographics and short bullet points. The PPI panel will be invited to contribute to the dissemination plan and all dissemination activities.

## Discussion

The incidence of stroke is continuing to rise ^38^. The impact of this disease can be severe and long-lasting for people with stroke, caregivers, and members of their family. Patient-centred, high-quality, and up-to-date research is important for health and social care practitioners working with people with stroke for an evidence-based approach to disease management. It is important to look at the long-term management of stroke for those who have left acute hospital care. Hall *et al.* (2022) have underlined the importance of PPI in stroke research and have hypothesised possible gaps in their recent protocol. They have noted that people with lived experience with stroke can provide thoughtful ad individual insights on research. By including key stakeholders in the area of stroke rehabilitation on research teams, it is hoped that emerging studies will become more accessible and have a holistic approach to study participants. Public and patient involvement is important when designing new services in healthcare ^39^. This study will create a stroke-specific PPI panel with members from the public, healthcare workers, and other researchers. The experiences and opinions of PPI panel will be examined. The processes, stages, and benefits of PPI in stroke research will be explored through the semi-structured interviews and focus groups. The results of this qualitative study will provide insight into the nature, extent, and impact of PPI in stroke rehabilitation research. Data analysis will be conducted with the input of PPI members and presented in visual and written form. Findings will be presented at academic conferences once published research journals suited to the area of public and patient involvement groups.

## Ethics and consent

Ethical approval is required for this study as data will be extracted and analysed from the meetings and interviews. PPI formation itself, however, does not require approval. Ethical approval has been granted by the Education and Health Science (EHS) ethics committee in the University of Limerick (2022_02_15_EHS) on 02/10/2023. Each invited member (people with stroke, carer/family member, healthcare professional) will be given an information letter and a consent form to be signed.

## Data Availability

No data are associated with this article.
