# Supplementary material for: Development and evaluation of a stroke research Public Patient Involvement Panel
Source: HRB Open Res. 2025 Apr 7;7:22. Originally published 2024 Apr 24. [Version 2] doi: 10.12688/hrbopenres.13838.2 (PMC12008718; doi:10.12688/hrbopenres.13838.2)
Supplement: Peer review history

# Reviewer report: Reviewer response for version 2

**DOI:** 10.21956/hrbopenres.15491.r46664

**Published:** 2025-04-18

**Authors:** Eithne Sexton

**Affiliations:** 1 School of Population Health, Royal College of Surgeons in Ireland, Dublin, Ireland

Thank you for the revisions, I am happy to approve the manuscript. 

 I noted a few minor errors for correction: 

 difficult In person will be carried out in disability-friendly environments that can be easily navigated and have close access to public transport.

 the Guidance for Reporting Involvement of Patients and the Public 2 (GRIPP2) checklist will be used as a guide for reporting patient and public involvement ^40^.

Is the study design appropriate for the research question?

Yes

Is the rationale for, and objectives of, the study clearly described?

Yes

Are sufficient details of the methods provided to allow replication by others?

Partly

Are the datasets clearly presented in a useable and accessible format?

Not applicable

Reviewer Expertise:

stroke, mixed methods, participatory research, epidemiology

I confirm that I have read this submission and believe that I have an appropriate level of expertise to confirm that it is of an acceptable scientific standard.

# Reviewer report: Reviewer response for version 2

**DOI:** 10.21956/hrbopenres.15491.r46665

**Published:** 2025-04-10

**Authors:** Phivos Phylactou, Marina Charalambous

**Affiliations:** 1 University of Western Ontario, London, Canada; 2 Rehabilitation Sciences, Cyprus University of Technology, Limassol, Cyprus

The authors have addressed the raised concerns, looking forward to the completed study.

Is the study design appropriate for the research question?

Yes

Is the rationale for, and objectives of, the study clearly described?

Yes

Are sufficient details of the methods provided to allow replication by others?

Partly

Are the datasets clearly presented in a useable and accessible format?

Not applicable

Reviewer Expertise:

PP: Cognitive Neuroscience, Brain stimulation; MC: Stroke Aphasia, PPI

We confirm that we have read this submission and believe that we have an appropriate level of expertise to confirm that it is of an acceptable scientific standard.

# Reviewer report: Reviewer response for version 1

**DOI:** 10.21956/hrbopenres.15162.r40736

**Published:** 2024-07-09

**Authors:** Eithne Sexton

**Affiliations:** 1 School of Population Health, Royal College of Surgeons in Ireland, Dublin, Ireland

Thank you for the opportunity to review the protocol for this study which will be a valuable contribution to the literature on PPI in stroke, which is a critically important area of study.

 It would be useful to elaborate on the extent to which the involvement will be “user-controlled”, as stated on p4. Does this mean that the research agenda will be fully driven by the PPI members? Or are there some restrictions to this based on funding? What research objectives will be feasible within the timeline of one year?

 It is clear that the authors have considered the inclusion of people with communication and other deficits, with a flexible involvement format, and input from a speech and language therapist (p4-5). It would be helpful to also consider this in the recruitment phase, and provide information materials that are aphasia-friendly.

 It was also not clear the extent to which there will be separate or combined meetings for the survivor/carer and the HCP/researcher group. There are likely advantages and risks to both approaches, and the best approach might be a mix of the two, or indeed for the PPI group members themselves to decide on this?

 On page 5 it is mentioned that a focus group will be held to “determine consensus” among groups. How will consensus be determined? Is consensus possible or desirable in this context? The differing experiences and perceptions of the groups are likely to be an important part of the analysis.  

 The authors state that they will use reflexivity to “acknowledge their biases and how these may affect the analysis of the interviews”. How specifically will the authors engage with reflexivity? Some more information on the methodological and theoretical orientation underpinning the research would also be helpful.

 More specific information on how the PPI panel will participate in the analysis process would be useful. Will they be part of the coding team, or will themes be shared for feedback and discussion, or both?

Is the study design appropriate for the research question?

Yes

Is the rationale for, and objectives of, the study clearly described?

Yes

Are sufficient details of the methods provided to allow replication by others?

Partly

Are the datasets clearly presented in a useable and accessible format?

Not applicable

Reviewer Expertise:

stroke, mixed methods, participatory research, epidemiology

I confirm that I have read this submission and believe that I have an appropriate level of expertise to confirm that it is of an acceptable scientific standard, however I have significant reservations, as outlined above.

# Reviewer report: Reviewer response for version 1

**DOI:** 10.21956/hrbopenres.15162.r39867

**Published:** 2024-05-30

**Authors:** Phivos Phylactou, Marina Charalambous

**Affiliations:** 1 University of Western Ontario, London, Canada; 2 Rehabilitation Sciences, Cyprus University of Technology, Limassol, Cyprus

We have carefully reviewed the protocol by O’Reilly and colleagues, who plan to develop a PPI panel for stroke research. The authors also plan to evaluate this PPI panel through focus groups.

 We applaud the authors for their proposed project, as we strongly believe that PPI is vital for the future of stroke research. Below, we provide our feedback on the protocol, in confidence that our review will help strengthen the planned work.

*Notes:* (1) Our paging references are according to the PDF version of the protocol. (2) We were not able to locate Appendix A that was referred to in the protocol.

**Comments:**

 In p.3, the second sentence needs to be rephrased/proofread. It seems that there is an additional full stop which is not necessary and is also incomplete (e.g., ‘in health and social and’): “There is particular emphasis on the need to consider Public & Patient Involvement. in health and social and is a key pillar of high-quality research internationally.”

 In paragraph 2 of the Introduction, the authors mentioned that "Moreover, roughly 2/3 of people post-stroke have communication problems, with 1/3 having aphasia." Recent research confirms that people with aphasia are traditionally excluded from participating in research due to perceived communication difficulties (Charalambous et al., 2020; *Brain sciences*, https://doi.org/10.3390/brainsci10100688). Have you considered including people with aphasia in the PPI group, or do you intend to do so? Please specify.

 In p.3, the NIHR PPI definition is provided, but with no reference as to where this is retrieved from. Authors should provide a citation.

 In p.4, the authors describe their sampling plan, mentioning that the panel will consist of approximately 20 members (five researchers/social care professionals, five carers, 10 people with stroke experience). A thorough sampling plan in any study is of vital importance and can affect the rigour of the study. Further, following the guidelines for reviewing a study protocol, it is important to ensure that adequate information is provided to ensure the feasibility and replicability of the study. However, we are understanding in regards to the barriers and difficulties of recruiting for such panels. Our recommendation is that the authors describe their sampling plan in terms of minimum/maximum rather than approximate. E.g., what is the minimum number of members (in each stakeholder group) that you will consider adequate to proceed with the panel? What is the maximum that you will be willing to recruit? Being transparent about the minimum and maximum sample will enhance the study’s rigour and ensure that the authors followed their protocol in the next stage.

 In p.4, it is stated that "‘User control’ is the highest level of involvement and it comprises the patients, members of the public, caregivers and/or family members organizing and deciding on the research agenda and recruiting researchers to work alongside them. This is the level that will be used for this PPI panel.” Please explain why the formation of the "PPI advisory group" was selected compared to "user control", which is an active and equal PPI partnership, as stated in the paper (McMenamin et al., 2022; *International Journal of Speech-Language Pathology*, https://doi.org/10.1080/17549507.2022.2075465).

 Also on p. 4, the authors state, “The formation of a PPI panel for stroke research will be supported by the guidelines developed from the INVOLVE framework.” Please consider the PAOLI framework (Charalambous et al., 2023; *Research Involvement and Engagement*, https://doi.org/10.1186/s40900-023-00484-9 ) on the PPI of people with aphasia and communication challenges after stroke as a guide for democratic and meaningful involvement.

 In p.4 (Panel involvement section), the authors mention that the meetings will take place between September 2023 and September 2024. Given that this is a protocol, I assume that this is a typo, as the panels cannot have taken place yet.

 In p.4 the authors describe that online meetings will be offered ‘as appropriate and according to the participant’s preference’. It is unclear what the purpose of these online meetings are. The authors should elaborate on their purpose. Additionally, the authors state that these meetings will be separate between individuals with stroke experiences/carers and healthcare professionals and researchers. This makes us a bit sceptical, considering the INVOLVE framework. For example, as proposed by INVOLVE (and outlined by the authors), part of PPI is about working together. This separation of meetings does not promote this collaboration and may also lead to miscommunication or bias towards one meeting group over the other. On a similar note, it is unclear if this separation is only for the online meetings or for the in-person meetings, too. Authors should be explicit if such a separation will be done (or not) during the in-person meetings.

 In p.4, “To ensure PPI members feel comfortable to share their opinions and experiences, separate meetings with be held with people with stroke and their caregivers, and the healthcare professionals and researchers.” Replace ‘with’ with ‘will’ for ‘separate meetings with be held’.

 In p.5 (Design of the qualitative study section), the authors note that they anticipate 4-6 participants in each stakeholder group for their focus group. This is not in line with their sampling plan description (i.e., five researchers/social care professionals, five carers). We invite the authors to follow a similar approach to our earlier comment on the sampling plan, where they describe the potential sample in terms of minimum/maximum. Further, considering that these will be done following a year of participation in the PPI panel, the authors should take into consideration potential drop-out of withdrawal from the study, in addition to the voluntary participation in the focus group that the authors already describe. Additionally, we suggest that the authors offer a contingency plan, where interviews will be conducted instead of the focus groups, in case they have an inadequate sample to conduct a focus group. As is, their current plan seems risky. Specifically, if they only have 5 researchers and 5 carers, dropping out and/or opting out rates, may restrict the authors from having an adequate focus group sample.  

 In p.5 (Qualitative data analysis section) second paragraph, second sentence start with a lowercase ‘the’ instead of a capitalised ‘The’.

 In p.6 ‘Hall et al., (2022)’ is not referenced in the references (unless this is a typo and the authors are referring to their ref. #17 which is referenced as a 2021 study).

 In p.6 “They have noted that people with lived experience with stroke can provide thoughtful ad individual insights on research”, ‘ad’ should be corrected to ‘and’.

 The current form of the protocol has two ‘ethics’ sections, one of which can be removed.

 Our understanding from the ‘Design of the qualitative study’ section is that the authors plan to conduct interview guided focus groups. However, in the ‘Qualitative data analysis’ section, the authors report that they will analyse ‘the focus group and interviews’. As such, it is currently unclear whether the authors plan to conduct focus groups and interviews or just (interview guided) focus groups. This should be made clear in the protocol to avoid confusion, and taken into consideration with our earlier comment about interviews serving as a contingency plan if required.

 A similar panel has been recently developed for stroke patients with aphasia (Charalambous et al., 2023; Research Involvement and Engagement, https://doi.org/10.1186/s40900-023-00484-9). Considering the large overlap with the authors’ planned work, the authors should discuss how this earlier work is related to their PPI panel, and how their PPI panel goes beyond this earlier work.

 Additionally, it would be helpful to add a paragraph in the methods section specifically explaining the following:

 - How were the advisory groups created? It seems that the advisory group will not participate equally in the project.

 - Did the PPI partners play any role in formulating the research questions and deciding on the design of the study?

 - What participatory methods are going to be employed in the study?

 - How will the authors approach the PPI partners in all advisory groups to avoid tokenism?

 - What are the obligations of the “PPI advisory group” in the research team?

 - What are the contributions expected from the “PPI advisory group” in each phase of the project? Please briefly explain how involving PPI will improve the study's impact and the implementation of the results.

 We hope this information will promote the transparent documentation of PPI and especially patient advisors’ contributions.

Is the study design appropriate for the research question?

Yes

Is the rationale for, and objectives of, the study clearly described?

Yes

Are sufficient details of the methods provided to allow replication by others?

Partly

Are the datasets clearly presented in a useable and accessible format?

Not applicable

Reviewer Expertise:

PP: Cognitive Neuroscience, Brain stimulation; MC: Stroke Aphasia, PPI

We confirm that we have read this submission and believe that we have an appropriate level of expertise to confirm that it is of an acceptable scientific standard, however we have significant reservations, as outlined above.

## References

[1] : Distinctions and blurred boundaries between qualitative approaches and public and patient involvement (PPI) in research. *Int J Speech Lang Pathol* .2022;24(5) : 10.1080/17549507.2022.2075465 515-526 10.1080/17549507.2022.2075465 35762365

[2] : Are People with Aphasia (PWA) Involved in the Creation of Quality of Life and Aphasia Impact-Related Questionnaires? A Scoping Review. *Brain Sci* .2020;10(10) : 10.3390/brainsci10100688 10.3390/brainsci10100688 33003493 PMC7600175

[3] : The development of the People with Aphasia and Other Layperson Involvement (PAOLI) framework for guiding patient and public involvement (PPI) in aphasia research. *Res Involv Engagem* .2023;9(1) : 10.1186/s40900-023-00484-9 74 10.1186/s40900-023-00484-9 37658465 PMC10474738
